# Dr. John H. Miller

**Published:** 1911-12

**Authors:** 


DR. JOHN H. MILLER of Niagara Falls died of apoplexy, November 10, 1911. He was a graduate of Bellevue, 1896, and was 50 years old.

